# Work stress trends in Germany: stable qualitative work overload but rising quantitative work overload across socio-demographic groups in a repeated cross-sectional study

**DOI:** 10.1186/s12889-025-25898-w

**Published:** 2025-12-16

**Authors:** Johannes Beller, Julia Graßhoff, Batoul Safieddine, Jelena Epping, Siegfried Geyer

**Affiliations:** 1 Hannover Medical School, Carl-Neuberg-Str. 1, 30625 Hannover, Germany; 2 HMU Health and Medical University Campus Düsseldorf/Krefeld, Kaistraße 16-16a, Düsseldorf, 40221 Germany

**Keywords:** Workload, Work overload, Work stress, Work, Health, Trends

## Abstract

**Background:**

The adverse effects of perceived excessive workload (work overload), as one major source of work stress, on health and work performance are well-documented; however, studies that examine how work overload has changed over time are lacking. This study aims to examine the time trends in work overload across and within socioeconomic groups in Germany.

**Methods:**

Data from the BIBB/BAuA Employment Surveys of 2006, 2012, and 2018 are used, with response rates between 44% and 14%. Work overload is operationalized by two indicators: qualitative overload (overload due to work difficulty) and quantitative overload (overload due to work amount). Logistic regression models are employed to examine the changes in work overload over time and by socioeconomic subgroup.

**Results:**

The results indicate that qualitative overload remained relatively stable over time, with minor increments among workers with a lower educational level. Conversely, quantitative overload increased substantially in the entire sample and in all subgroups. The rise in quantitative overload was especially pronounced among workers with a lower educational level, who also reported increasing levels of qualitative overload.

**Conclusions:**

These findings show that perceived work overload has increased over time in Germany, but mostly regarding work quantity. The study highlights the public health need for managing workload to alleviate stress and enhance health and well-being in the workplace.

**Supplementary Information:**

The online version contains supplementary material available at 10.1186/s12889-025-25898-w.

## Background

Workload refers to the amount and complexity of work that a worker has to perform within a given time frame [1, 2]. Perceived excessive workload, also called work overload, occurs when the workload exceeds the worker’s available resources, such as time, energy, knowledge or skills. Work overload can be classified into two types: quantitative and qualitative [3]. Quantitative overload refers to having too much amount of work to complete, while qualitative overload refers to having work that is too difficult for the worker’s level of knowledge and skills. Thus, an individual experiencing qualitative overload may have a manageable amount of work but find the work tasks too difficult and complex. Conversely, an individual experiencing quantitative overload may find the work tasks manageable but may have a too high amount of work to do.

Work overload is associated with negative health outcomes [4]. Several studies have shown that work overload predicts psychological distress, including increased stress, fatigue, feelings of burnout and depression [2, 5, 6]. The physiological effects of chronic work overload are also significant [7–9]. When faced with excessive workload, workers exhibit heightened cardiovascular responses, including elevated blood pressure and heart rate [7, 10]. Overload also alters the Hypothalamic–pituitary–adrenal axis functioning, causing for example an increased release of cortisol [11, 12]. Consequently, work overload is associated with various negative health- and work-related outcomes including hypertension, diabetes, sickness absence, work injuries and mortality [13–17]. More generally, as a major source of work stress, work overload is likely associated with a broad spectrum of stress-related diseases. Chronic stress exposure triggers allostatic overload, where repeated activation of stress responses leads to dysregulation across multiple biological systems [18]. This dysregulation contributes to numerous pathological conditions, most notably cardiovascular diseases, immunosuppression, metabolic disorders, mental health conditions and generally accelerated cellular aging [18].

Understanding how work overload changes over time is therefore crucial for several reasons. First, knowledge about temporal trends in work overload can help identify whether occupational health risks are increasing or decreasing in the population, allowing for targeted interventions and policy development. Second, if work overload is increasing over time, this could partly explain observed trends in related health outcomes such as rising rates of mental health issues and chronic diseases in working populations. Third, examining trends across different socio-demographic groups can improve the understanding of health inequalities related to work. Finally, understanding these trends can help organizations and policymakers anticipate future challenges and develop preventive strategies to protect worker health.

Some previous studies have examined time trends in related constructs and found increases over time regarding for example work intensity and work stress [19–22]. For example, Maume and Purcell documented rising work intensity among American workers from the 1977 to 1997 [19]. Similarly, Rigó and colleagues found increases in various work stressors across European countries from 2005 to 2015 [20]. Their comprehensive analysis of 15 European countries from 1995 to 2015 revealed that work stress factors generally increased during this period, primarily driven by rising psychological demands. Notably, they found that workers in lower-skilled occupations not only experienced higher levels of job strain and effort-reward imbalance but also showed steeper increases in job strain compared to those in higher-skilled positions. A subsequent study by the same authors demonstrated that this trend varied significantly based on countries’ labor market policies - with a 10% increase in job strain from 1995 to 2015 in countries with low labor market policy spending, while countries with higher policy investments showed more stable trends [21]. These trends have been attributed to factors such as technological changes enabling constant connectivity, increased global competition, and economic pressures for greater productivity [23].

Given these documented trends of increasing work intensity and job demands, we hypothesize that work overload has likely increased over time as well. Specifically, as workers face greater time pressure, multitasking requirements, and performance expectations, their perception of having too much work (quantitative overload) or work that is too difficult (qualitative overload) may increase as well. However, studies on time trends in work overload remain lacking. Work overload is related to the broader concepts of work intensity and work stress examined in most previous studies. Work intensity generally refers to the pace and effort of work, while work stress generally refers to the overall distress related to work and adverse work conditions, such as an unfavorable relation of effort to reward [3, 24]. In contrast, work overload specifically refers to the evaluation that job demands, either qualitative (task difficulty) or quantitative (amount of work), exceed a worker’s capacity or available time [4]. While increasing work intensity may contribute to work overload, overload focuses on the mismatch between demands and capacity. By examining trends in work overload specifically, this study provides novel insights beyond existing research on broader work stress constructs.

Moreover, examining trends in work overload across different sociodemographic groups seems important for understanding potential social inequalities in work health risks. Previous research has shown that work stressors and health outcomes often differ by sociodemographic factors such as age, gender, education level, and occupation type [25–30]. For example, workers with lower education levels may be more vulnerable to adverse effects of work stress on health [31, 32]. Additionally, examining differential trends can reveal whether certain groups are experiencing worsening conditions over time relative to others. Therefore, to comprehensively analyze trends in work overload it seems important to also examine trends stratified by key sociodemographic variables to uncover potentially diverging patterns across subgroups.

The aim of the current study is thus to fill these gaps in the literature by analysing the data from the BIBB/BAuA Employment Surveys of 2006, 2012, and 2018. We ask: “How has work overload changed over time?”. Based on the literature showing increases in related constructs like work intensity and job demands, we hypothesize that work overload has increased over time. This study will provide important insights into how a key job stressor has evolved in recent years, with implications for employee wellbeing and organizational practices.

## Methods

### Sample

Data were drawn from the 2006, 2012, and 2018 cross-sectional BIBB/BAuA Employment Surveys. The sample frame of all BIBB/BAuA Employment Surveys includes German-speaking employees in Germany who are at least 15 years old and work at least ten hours per week. The BIBB/BAuA Employment Surveys are conducted as a collaboration between the German Federal Institute for Vocational Education and Training (BIBB) and the German Federal Institute for Occupational Safety and Health (BAuA). These surveys constitute the most comprehensive and important labor market surveys in Germany. They are regularly used as a resource for monitoring changes in for example qualification requirements, working conditions, and occupational health across the German workforce. The surveys’ particular strength lies in their detailed information on occupations, job tasks, and working conditions. These surveys have been conducted periodically, with the latest available survey having been conducted in 2018 [33–35]. The latest wave of the survey was conducted in 2024, but the Scientific Use File for this data was not yet available for analysis at the time of our study. To be eligible for the survey, participants must meet the following criteria: they are 15 years of age or older, work for at least 10 h per week, and have an adequate knowledge of the German language.

The sampling procedure involved a random-digital-dialing approach for landline (in 2006 and 2012) and both landline and mobile numbers (in 2018), resulting in response rates of 44.0% in 2006, 44.3% in 2012 and 13.9% in 2018 based on the number of interviews related to the eligible addresses (the lower response rate in 2018 is largely due to the inclusion of random mobile phone numbers in 2018, which required a much larger gross sample due to the high proportion of invalid mobile numbers). The interviews, which lasted about 40 min on average, focused on sociodemographic variables, work activities, working conditions, and health. Participants provided informed consent, and all procedures were conducted in accordance with German law and the 1964 Helsinki declaration and its later amendments. The BAuA ethics committee approved the surveys, with the latest approval being EK007_2017 on January 9, 2017. After listwise deletion of participants with missing values (*n* = 2,034, about 3% of the original sample size), the final sample of *N* = 58,014 participants (N_2006_ = 19,264, N_2012_ = 19,355, N_2018_ = 19,395) resulted.

### Measures

The operationalization of qualitative overload was based on the following questions: “Do you feel usually up to the requirements of your occupational knowledge and skills, rather overchallenged or rather underchallenged?” (translated from German). Participants could choose to respond with “usually up to the requirements”, “rather overchallenged”, or “rather underchallenged”. The same question was repeated for quantitative overload: “And do you usually feel up to the demands of the amount of work or the workload, rather overchallenged or rather underchallenged?”. The response options for both variables were coded as follows: “feeling overchallenged” (1) vs. “not feeling overchallenged (other responses)” (0). This resulted in the two dependent binary variables qualitative overload (feeling overchallenged regarding one’s occupational knowledge and skills) and quantitative overload (feeling overwhelmed regarding the amount of workload).

The covariates were operationalized as follows. Age: Age was operationalized in years. Gender: Gender was operationalized as male or female. Working hours were operationalized as “part-time” if the average weekly working hours reported by participants was lower than 30 and operationalized as “full-time” if the average weekly working hours reported by participants was equal to or higher than 30. Occupation: Participants were classified into four occupational groups based on the International Standard Classification of Occupations (ISCO): White-Collar High-Skilled (ISCO: 1 Managers, 2 Professionals, 3 Technicians), White-Collar Low-Skilled (ISCO: 4 Clerical Support Workers, 5 Service Workers), Blue-Collar High-Skilled (ISCO: 6 Skilled Agricultural workers, 7 Craft workers), and Blue-Collar Low-Skilled (ISCO: 8 Machine Operators, 9 Elementary Occupations). Education: Participants were categorized into three educational attainment levels based on their highest school educational attainment of “High” (at least a “Abitur oder Hochschulreife”, which is equivalent to a general qualification for university entrance or advanced high school diploma), “Intermediate” (“Mittlere Reife”, akin to an intermediate school leaving certificate or secondary education diploma), and “Low” (up to a “Hauptschulabschluss”, comparable to a basic secondary school leaving certificate).

### Data analysis

First, descriptive statistics of all variables are reported based on the unweighted data to describe the samples across time periods. Differences in work overload across sociodemographic subgroups were analyzed via chi-square tests and Cramer’s V effect size [36]. Then, to determine population trends, weighted logistic regression analyses are conducted predicting qualitative or quantitative overload via age (in years), gender (0 = male; 1 = female) and time period (scaled as a metric variable between 0 and 1 with 2006 = 0, 2012 = 0.5, and 2018 = 1, such that the effect of time period can be interpreted as the change between 2006 and 2018; however, similar results are obtained when comparing the waves directly). All regression analyses, including the subgroup analyses, were adjusted for age and gender.

Trends were also analysed in stratified samples according to age, gender, education, working time and occupational group. To formally test whether trends differed significantly between sociodemographic groups, we conducted additional logistic regression analyses including interaction terms between time period and each sociodemographic variable (age group, gender, education level, working hours, and occupation). As some have cautioned regarding the interpretability and comparability of odds ratios, we also report predictive probabilities of reporting overload in 2006 compared to 2018, based on the same logistic regression analyses, adjusting for age and gender [37].

In addition to our primary analyses, we conducted a supplementary analysis predicting work overload controlling for all covariates (age, gender, education, occupation, and working hours) simultaneously to examine how these factors might influence the observed trends in work overload. This supplementary analysis provides insights into the robustness of our findings and provides information on what trends would have been expected to occur statistically if no differences over time would have occurred regarding these covariates.

All regression analyses are weighted according to the design weights provided by the BIBB/BAuA Employment Surveys. These weights are intended to adjust for the sampling design and non-response to improve the representativeness of the trend estimates. It’s important to note that while these weights were used in the regression analyses, they were not applied to the descriptive statistics.

## Results

As depicted in Table 1, participants were on average 44.89 years old (SD = 11.12), worked 38.55 h per week (SD = 12.18), and 41.3% had a high level of education. About half of them were female (50.3%) and most of them belonged to the high-skilled white-collar occupation group (57.7%). The overall prevalence of qualitative and quantitative overload was 3.9% and 20.7%, respectively. As depicted in Table 2, prevalence of qualitative work overload did not differ significantly according to age or gender. However, there were significant differences according to education, working hours, and occupation. Qualitative work overload was more prevalent among workers with low educational attainment (5.3%), full-time workers (4.0%), and workers in the blue-collar high-skilled (4.5%) as well as blue-collar low-skilled occupations (4.4%). Prevalence of quantitative work overload significantly differed according to age, gender, education, working hours, and occupation. Quantitative work overload was more prevalent among workers aged 40 or older (21.1%), female workers (22.6%), workers with high educational attainment (23.3%), full-time workers (22.2%), and among workers in the white-collar high-skilled occupations (23.6%). Effect sizes of these socio-economic differences were however small, Cramer’s *V*s < 0.10 for the overall analysis and in every time period (Appendix Table A1).

**Table 1:** Work overload and sociodemographic characteristics

		Stratified by Time Period		
	Overall	2006	2012	2018
n	58,014	19,264	19,355	19,395
Overload: Qualitative (%)	3.9	3.9	3.6	4.1
Overload: Quantitative (%)	20.7	18.5	19.7	24.0
Age (mean (SD))	44.89 (11.12)	41.32 (10.42)	46.06 (10.71)	47.26 (11.31)
Gender = Female (%)	50.3	48.7	52.5	49.8
Education (%)				
High	41.3	35.2	35.1	53.7
Intermediate	37.2	39.2	40.5	31.8
Low	21.5	25.6	24.4	14.5
Working Hours (mean (SD))	38.55 (12.18)	38.90 (12.90)	38.51 (12.04)	38.25 (11.54)
Occupation (%)				
WC-HS	57.7	53.8	55.1	64.2
WC-LS	19.8	21.1	21.4	17.0
BC-HS	12.2	14.1	12.7	9.8
BC-LS	10.3	11.0	10.8	9.0

*N* sample size, *SD* Standard Deviation, *WC-HS* White-Collar High Skilled, *WC-LS* White-CollarLow Skilled, *BC-HS* Blue-Collar High Skilled, *BC-LS* Blue-Collar Low Skilled

*Notes*. Unweighted estimates

**Table 2:** Work overload across sociodemographic subgroups

Subgroup	Qualitative Work Overload	Quantitative Work Overload
All	3.9%	20.7%
Age	(*p* =.293, *V* = 0.004)	(*p* =.003, *V* = 0.012)
Age < 40	4.0%	20.0%
Age > = 40	3.8%	21.1%
Gender	(*p* =.619, *V* = 0.002)	(*p* <.001, *V* = 0.047)
Male	3.8%	18.8%
Female	3.9%	22.6%
Education	(*p* <.001, *V* = 0.030)	(*p* <.001, *V* = 0.041)
High	3.1%	23.3%
Intermediate	3.9%	19.8%
Low	5.3%	17.4%
Working Hours	(*p* = < 0.001, *V* = 0.018)	(*p* <.001, *V* = 0.077)
Part-time	3.1%	14.2%
Full-time	4.0%	22.2%
Occupation	(*p* <.001, *V* = 0.010)	(*p* <.001, *V* = 0.047)
WC-HS	3.7%	23.6%
WC-LS	3.6%	17.3%
BC-HS	4.5%	16.9%
BC-LS	4.4%	16.1%

Unweighted estimates

*V* Cramer’s V, *WC-HS* White-Collar High Skilled, *WC-LS* White-Collar Low Skilled, *BC-HS* Blue-Collar High Skilled, *BC-LS* Blue-Collar Low Skilled

*P*-values are based on chi-squared tests, testing whether the prevalence of overload differed acrossgroups regarding the respective socioeconomic indicator

Table 1 also shows the changes in the variables over time from 2006 to 2018. The average age of the participants increased by 5.94 years, while the average working hours decreased by 0.65 h. The proportion of participants with high education increased by 18.5% points, while the proportion of those with low education decreased by 11.1% points. The gender distribution remained relatively stable, with a slight increase in the percentage of female participants in 2012. The occupational structure changed significantly, with an increase in the high-skilled white-collar group by 10.4% points and a decrease in the other groups by 2.3 to 5.3% points. The prevalence of qualitative overload fluctuated slightly, with a minimum of 3.6% in 2012 and a maximum of 4.1% in 2018. The prevalence of quantitative overload increased steadily, reaching a peak of 24.0% in 2018, which was 5.5% points higher than in 2006.

### Time trends

**Fig. 1:**
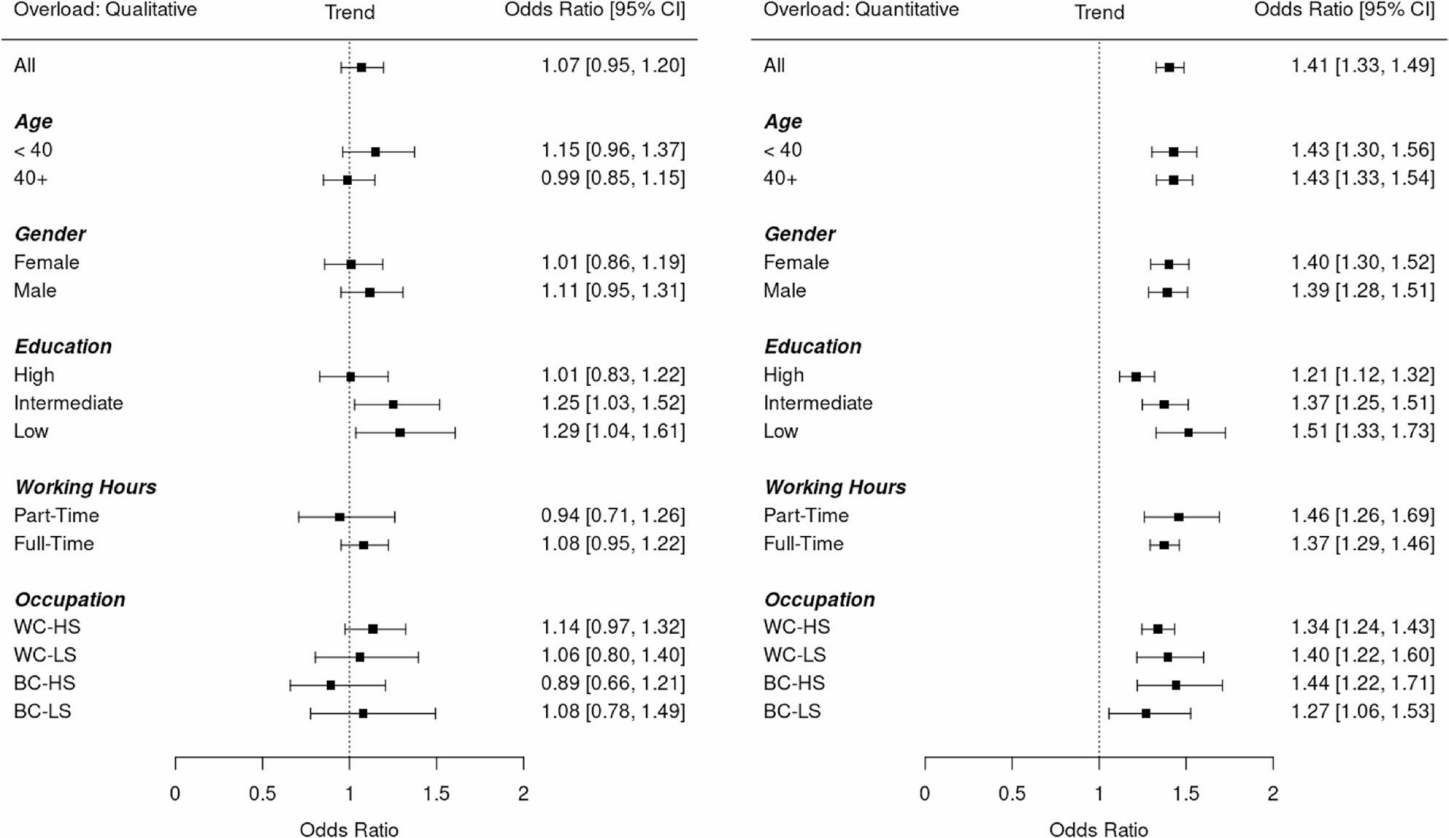
Time trends in overload. Notes. Depicted are the odds ratios for time period predicting overload controlling for age and gender via logistic regression using weights. For each subgroup a separate logistic regression analysis was conducted. WC-HS = White-Collar High Skilled; WC-LS = White-Collar Low Skilled; BC-HS = Blue-Collar High Skilled; BC-LS = Blue-Collar Low Skilled

Next, as depicted in Fig. 1, several regression analyses were used to study trends in qualitative and quantitative work overload in the general sample and in socioeconomic subgroups. As discussed, qualitative overload refers to the perception that work is too difficult, while quantitative overload relates to having too much work. For qualitative overload, the results showed that the odds of feeling overloaded did not change significantly over time in the general sample (OR = 1.07, 95%-CI: [0.95, 1.20]) and most subgroups, meaning there was only a general non-significant 7% increase in the likelihood of experiencing qualitative overload. Only in the case of workers with a lower or intermediate educational attainment small increases over time were observed (OR = 1.29, 95%-CI: [1.04, 1.61] and OR = 1.25, 95%-CI: [1.03, 1.52], respectively), indicating that workers with lower education were 29% more likely over time to experience qualitative overload. When controlling for all covariates (age, gender, education, working hours, occupation) simultaneously, a significant small increase of qualitative workload over time was observed for the whole sample (OR = 1.19, 95%-CI: [1.06, 1.34]). For qualitative overload, we found a significant interaction only for education level, indicating that the trends over time differed across educational groups (education_high_ vs. education_low_: *p* =.050).

For quantitative overload, the results showed that the odds of feeling overloaded increased significantly over time in the general sample (OR = 1.41, 95%-CI: [1.33, 1.49]) and in all the subgroups, meaning there was a statistically significant and substantial 41% increase in the likelihood of experiencing quantitative overload over time. The largest relative increase in the odds of feeling quantitatively overloaded was observed among those with a lower educational attainment (OR = 1.51, 95%-CI: [1.33, 1.73]), indicating that workers with lower education were 51% more likely over time to experience quantitative overload. When controlling for all covariates, a significant increase of quantitative workload over time was still observed for the whole sample (OR = 1.37, 95%-CI: [1.30, 1.46]), demonstrating that the increase in quantitative overload cannot be explained by changes in demographics. For quantitative overload, we also found a significant interaction only for education level, indicating that the trends over time differed across educational groups (education_high_ vs. education_low_: *p* =.021), with lower educated workers experiencing more pronounced increases in workload over time compared to higher educated workers.

**Fig. 2:**
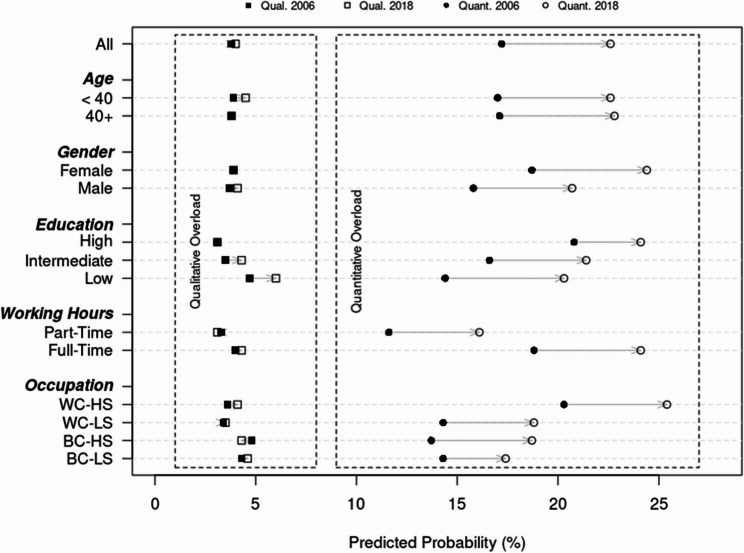
Predicted probabilities of qualitative work overload and quantitative work overload over time.Notes. Depicted are the predicted probabilities of reporting qualitative work overload (squares) or quantitative work overload (circles) for 2006 (filled squares and circles) and 2018 (unfilled squares and circles). For each subgroup a separate analysis was conducted. WC-HS = White-Collar High Skilled; WC-LS = White-Collar Low Skilled; BC-HS = Blue-Collar High Skilled; BC-LS = Blue-Collar Low Skilled

In terms of predicted probabilities, as visualized in Fig. 2, the likelihood of experiencing qualitative overload remained relatively stable between 2006 and 2018, being around 4% for most groups, meaning that at any given time, approximately 4% of workers reported their work being too difficult, with slightly higher probabilities and trends observed for those with lower education (increased from about 4% in 2006 to 6% in 2018). In contrast, the predicted probability of experiencing quantitative overload showed more variation and substantial increases across all groups, rising from approximately 17% in 2006 to 23% in 2018, meaning that nearly one in four workers reported having too much work to do in 2018, a substantial increase from approximately one in six workers in 2006. Similar to the main results, differential increases in quantitative overload were mainly observed regarding education, where those with lower education experienced stronger increases in predicted probabilities as compared to those with a higher education. Taken together, these findings suggest that while qualitative work overload has remained largely stable or slightly increased over time, quantitative work overload has become much more prevalent across socioeconomic groups in recent years, pointing to a perceived intensification of work rather than increasing complexity.

As a robustness analysis, testing whether our findings hold up when looking at smaller time intervals, the year-by-year comparisons of qualitative work overload show that between 2006 and 2012, overload remained relatively stable, while it increased very slightly from 2012 to 2018 (please see the Appendix Figure A1). Regarding quantitative work overload, it generally increased from 2006 to 2018, although the increase from 2012 to 2018 was much more substantial, indicating an acceleration in perceived work intensification during the most recent years. These specific year comparisons thus align with the overall trends, where a general increase in quantitative work overload and a relative stability or very slight increase of qualitative work overload was observed.

## Discussion

We examined the time trends in work overload across various socioeconomic groups in Germany, utilizing data from the BIBB/BAuA Employment Surveys spanning 2006, 2012, and 2018. Our aim was to understand how perceived work overload has changed over time, both in terms of work difficulty (qualitative overload) and work amount (quantitative overload). We found that qualitative overload has remained relatively stable over the years, with only slight significant increases observed among workers with lower educational levels. However, quantitative overload showed a significant rise across the entire sample and within all socioeconomic subgroups. This increase was particularly marked among lower-educated workers, who also reported a rise in qualitative overload. These trends occurred alongside an aging workforce that was more highly educated and more likely to work in high-skilled white-collar occupations. This aligns with general population trends in Europe, where demographic aging, educational expansion, and a shift towards knowledge-based work have been well documented [38].

These results confirm previous studies indicating a general rise in work stress. For example, Maume and Purcell documented significant increases in work intensification among American workers [19]. Similarly, Rigó and colleagues found that work stress generally increased across 15 European countries from 1995 to 2015, with the increase primarily driven by rising psychological demands [20]. Their research further revealed that workers in lower-skilled occupations showed stronger increases in job strain compared to those in higher-skilled positions—a pattern partially reflected in our findings regarding differential increases in work overload by education level. In contrast to the negative trends in our studies, some previous studies have also found positive developments. For example, Beller and colleagues found improvements in several physical working conditions in Germany alongside an aging and upskilling workforce [22]. Taken together, the current state of the literature thus indicates adverse trends in work stress might mostly apply to the quantitative psychosocial domain. However, going beyond previous studies, we provide the first analysis focusing specifically on trends in work overload. The results suggest that the increases in work overload might be one specific contributor to increases in general work stress. Further studies are needed to replicate these trends.

### Implications

From a practical perspective, the study results suggest that quantitative work overload is a wide-spread and growing occupational health problem in Germany, affecting over 20% of workers across socioeconomic groups. The prevalence of qualitative work overload was much lower at around 4%, suggesting this is less of a widespread concern. While our study did not directly examine health outcomes, prior research has linked work overload to negative consequences for worker health and performance [4]. Based on this evidence and the current study results, there is a need for prevention and intervention strategies to reduce work overload and its negative consequences on health and work performance. Such strategies could for example include implementing regulations that limit excessive work demands [39], and providing workers with more control and support [40]. However, for such strategies to be implemented first more information on the concrete reasons for the substantial increases in quantitative and the more attenuated increases in qualitative work overload is needed. These strategies could be especially beneficial for workers with a lower educational level, who were most significantly affected by increasing work overload over time and might be most vulnerable to its adverse effects [41]. Moreover, workers who are employed in white-collar high-skilled jobs, such as managers, professionals, and technicians, could also benefit from these strategies, as the prevalence of quantitative overload was highest in this occupational group.

Regarding the implications for the most relevant stakeholders, employers should implement workload assessment protocols to regularly monitor employee workload levels. This could include quarterly workload reviews integrated with performance evaluations to identify overtasked employees before health issues emerge. Organizations should also establish clear boundaries for work expectations, especially in white-collar high-skilled occupations where quantitative overload was found to be most prevalent. For workers developing skills in boundary-setting and time management to better manage increasing quantitative workload demands seem increasingly important. Workers with lower educational attainment, who showed increased vulnerability to both types of work overload over time, would particularly benefit from seeking opportunities for skill development. Additionally, our findings of increased work overload, particularly quantitative overload, suggest the need for policy interventions. Governments should consider implementing and enforcing regulations on maximum working hours and minimum rest periods, with particular attention to sectors showing high levels of quantitative overload. Policy makers could also develop educational and vocational training programs specifically designed to address the skill gaps contributing to qualitative overload among less educated workers. Finally, governments should continue to invest in research on the economic and public health impacts of work overload to better inform future policy decisions and provide incentives for organizations that implement effective workload management practices.

The study results also contribute to the literature on the changing world of work and health trends. The study provides empirical evidence that work overload has increased over time in Germany, but mostly regarding work quantity. This finding supports the notion that the modern work environment is increasingly characterized by high work intensity [23, 42]. Data from European work surveys show that average weekly working hours have gradually decreased across most European countries since the early 2000 s, dropping from approximately 38.6 to 36.6 h between 2005 and 2015 [43]. Our study corroborates this finding, showing a slight decrease in average weekly working hours over the study period. Interestingly, despite this reduction in work hours, workers report feeling more quantitatively overloaded. This suggests that the increase in perceived workload may be attributed to factors other than longer work hours, such as increased job demands or reduced resources. This phenomenon can be described as an “intensification paradox”: Employees work fewer hours but feel more overwhelmed by their workload.

The study also reveals that work overload prevalence varies by socioeconomic group. Interestingly, contrarian social inequalities were observed [25, 26, 44]. Whereas workers with a lower socioeconomic status were more affected by qualitative overload, the opposite held true for quantitative overload. Here, workers with a higher socioeconomic status were most affected by quantitative overload, in contrast to most other health risk factors. This pattern of qualitative overload among workers with lower socioeconomic status may be interpreted as a form of underqualification, where job demands exceed the worker’s knowledge and skills [45]. This interpretation aligns with our specific measure of qualitative overload, which asks participants about feeling overloaded due to occupational knowledge and skill requirements. The observed pattern may also reflect broader structural changes in the labor market [46]. For instance, the reduction in low-skilled job offers in many sectors could have contributed to a mismatch between available jobs and the skills of workers with lower educational attainment. This mismatch might increase the likelihood of these workers feeling qualitatively overloaded in their roles over time, as observed in the current study. Conversely, the higher prevalence of quantitative overload among workers with higher socioeconomic status might be attributed to increased job responsibilities and expectations in more skilled positions [47]. This finding also aligns with some of the literature on sedentary lifestyle, where also those working within white-collar high skilled occupations are most likely to be affected [48–50]. Therefore, those with more highly skilled occupations may face partly distinct health risks from those with a lower socioeconomic status.

Finally, the study results also have implications for explaining the health trends in younger and middle-aged adults, where increasing morbidity has been observed in recent years, in line with the expansion of morbidity hypothesis [51–56]. The current results suggest that increasing work overload might have contributed to this phenomenon, as work overload might have led to increased stress and the accompanying impairments in mental and physical health [4]. It might also be expected that the increased work overload could have also affected the work-life balance and work-family conflicts of workers, which should be investigated by future studies.

### Limitations and future research directions

The current study has several limitations that should be acknowledged. First, the study relies on self-reported measures of work overload, which may be subject to bias and social desirability effects [57]. Although perceived overload might be the most relevant indicator of workload from a health perspective, it would be desirable to complement it with more objective measures [1]. Second, the study uses a single-item measure of work overload for both qualitative and quantitative aspects, which may not capture the full complexity and multidimensionality of the construct. For example, in the current study only time trends regarding prevalence could be investigated. It also seems likely that work overload intensity has changed over time as well. A more comprehensive and validated scale of workload could thus provide more reliable and nuanced results for future studies [58, 59]. Third, the study does not account for the possible interactions between overload and other important aspects of work quality, such as social support and control, which might be investigated by future studies [60]. Fourth, our supplementary analysis, in which we controlled for additional socioeconomic factors, revealed a slightly attenuated but still significant increase in quantitative work overload over time and an enhanced and more significant increase in qualitative work overload over time. This suggests that while changes in these covariates such as education and occupation contribute to the observed trends, they do not fully explain the increase in work overload. The persistence of this trend and the emergence of a significant qualitative work increase after controlling for these factors underscores the pervasive nature of this phenomenon across various sectors and sociodemographic groups of the workforce. Future research could further explore the specific mechanisms through which societal and organizational changes contribute to increased work overload, beyond shifts in educational and occupational structures.

Fifth, although we used population-based samples and weighting in the regression analyses, the educational composition of the sample could be questioned. The participants were predominantly highly educated in the current study sample, which raises concerns about the representativeness of the findings for the general population. For example, about half of participants had a high educational level in our study of workers, whereas about 33% had the same in the general German population [61]. Given that education emerged as an important stratification variable for work overload trends in our results, future studies might further explain this topic, e.g. by using more fine-grained operationalizations of educational attainment like CASMIN [62]. This is particularly important due to the potential for non-response bias, especially in the latest wave of the survey [63]. If individuals with lower educational levels are increasingly less represented, there is a risk that the increasing trends in work overload could be underestimated in the current study. Future studies are needed focusing especially on less-educated and vulnerable worker populations. In a similar vein, an important limitation concerns the notable decline in response rates across survey waves, from 44.0% in 2006 to just 13.9% in 2018. While this decline is partially attributable to methodological changes (including the addition of mobile phone sampling in 2018), it raises concerns about potential non-response bias affecting our trend estimates. If non-response is systematically related to work overload (for instance, if highly overloaded workers are less likely to participate in later survey waves), our estimates of increasing work overload might be too conservative and the increasing trends in quantitative work overload might even be underestimated.

While our study provides findings on work overload trends in Germany, we also acknowledge that caution is needed in generalizing these findings to other national contexts. Work demands as well as workers’ resources and work perceptions are likely to vary across countries due to differences in labor policies, economic structures, and cultural factors [21]. Some broad trends in work intensification have been observed across many contexts, especially high-income countries [20, 23], suggesting our findings have wider relevance. However, the specific patterns and magnitudes of change in work overload likely differ between countries. Future cross-national comparative research is needed to examine how trends in work overload may vary across different national contexts. Such research could help identify which aspects of our findings reflect broader international trends versus Germany-specific developments.

### Conclusions

Despite these opportunities for future research, we were able to examine the time trends in work overload across and within socioeconomic groups, using a large and representative sample of workers from three waves of the BIBB/BAuA Employment Surveys. We found that work overload has increased over time, especially in terms of work quantity. These findings have important implications for public health and work design, as they suggest that work overload is a prevalent and growing problem in the labor market, and that it may contribute to the worsening health trends observed among middle-aged and younger adults. Policy responses to address these trends should include strengthening workload regulations and developing tailored interventions for vulnerable groups, particularly those with lower educational attainment. Organizations might benefit from implementing workload management systems that can identify excessive demands before they lead to health problems. Future studies should replicate and expand upon our results, using additional measures and by exploring the mediating and moderating factors that may covary with work overload across and within socioeconomic groups.

## Supplementary Information

Supplementary Material 1: Appendix

## Data Availability

This paper uses data from the BIBB/BAuA Employment Survey of the Working Population on Qualification and Working Conditions in Germany 2006, 2012 and 2018, dois: 10.7803/501.18.1.1.10, 10.7803/501.06.1.1.30. The data access is provided by the Data Research Centre of the Federal Institute for Vocational Training and Education (BIBB-FDZ) upon application.
