# Supplementary material for: Work stress trends in Germany: stable qualitative work overload but rising quantitative work overload across socio-demographic groups in a repeated cross-sectional study
Source: BMC Public Health. 2025 Dec 16;26:254. doi: 10.1186/s12889-025-25898-w (PMC12822222; doi:10.1186/s12889-025-25898-w)
Supplement: Supplementary file 1 — Supplementary Material 1: Appendix [file 12889_2025_25898_MOESM1_ESM.docx]

APPENDIX

Figure A1. *Time Trends in Overload Comparing Specific Years*


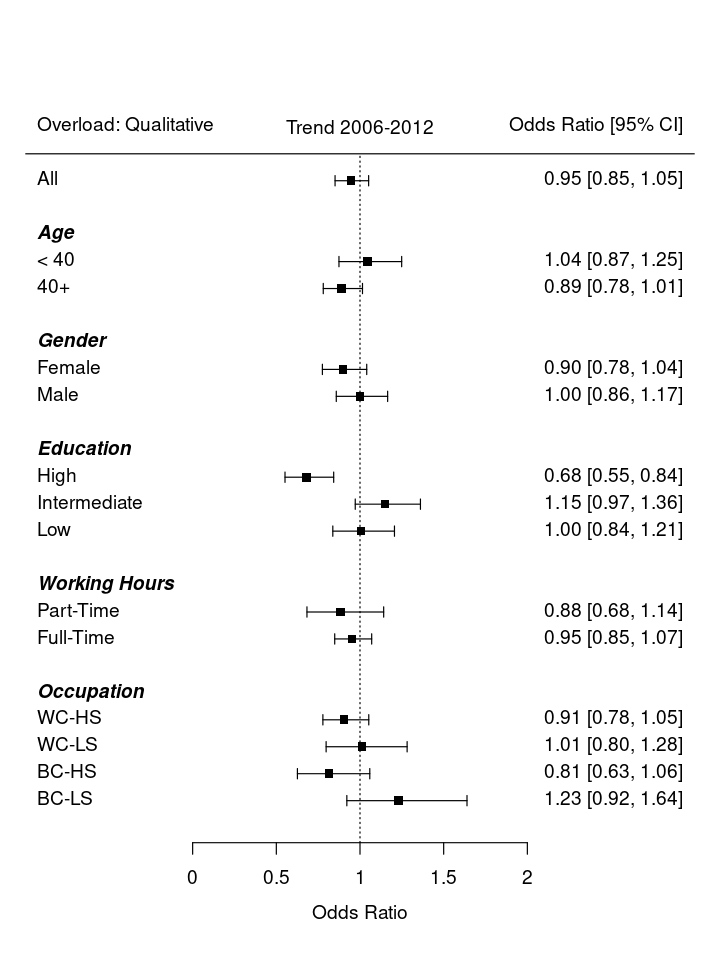

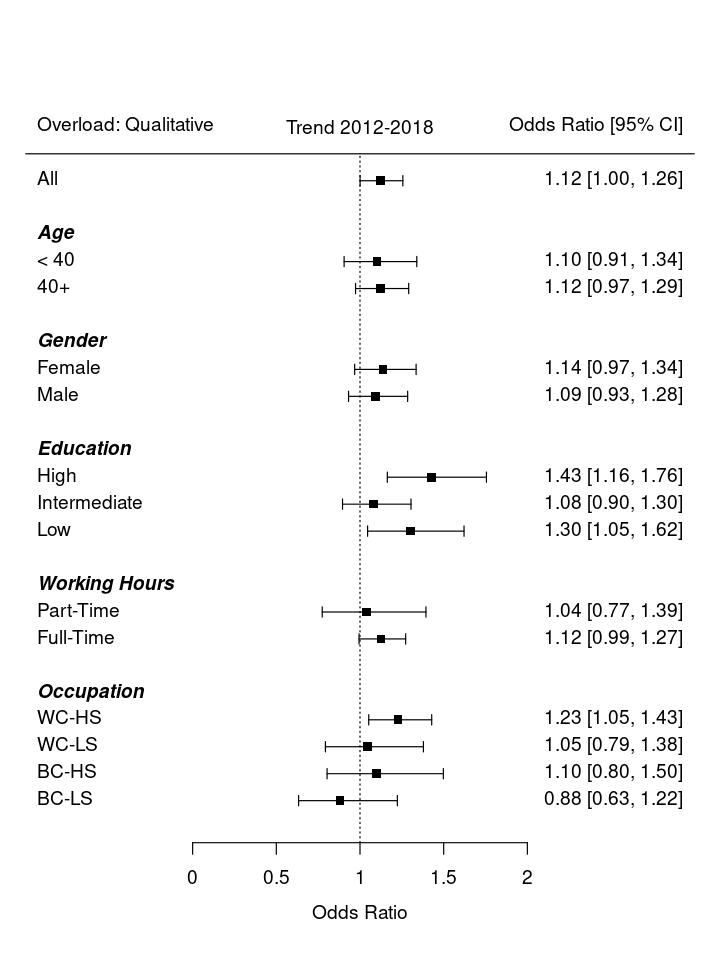


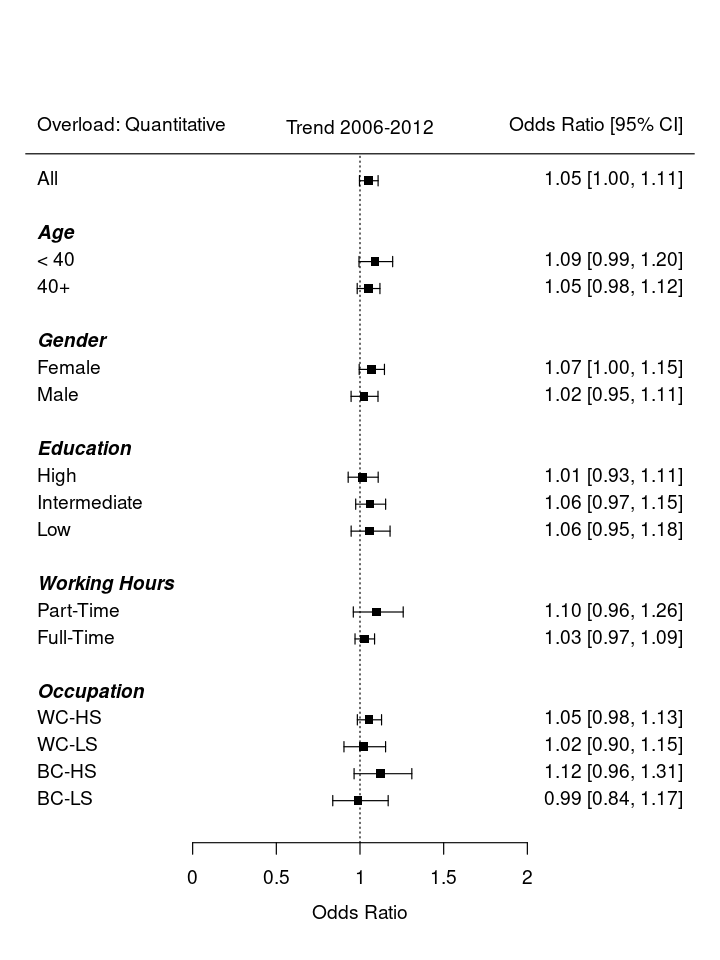

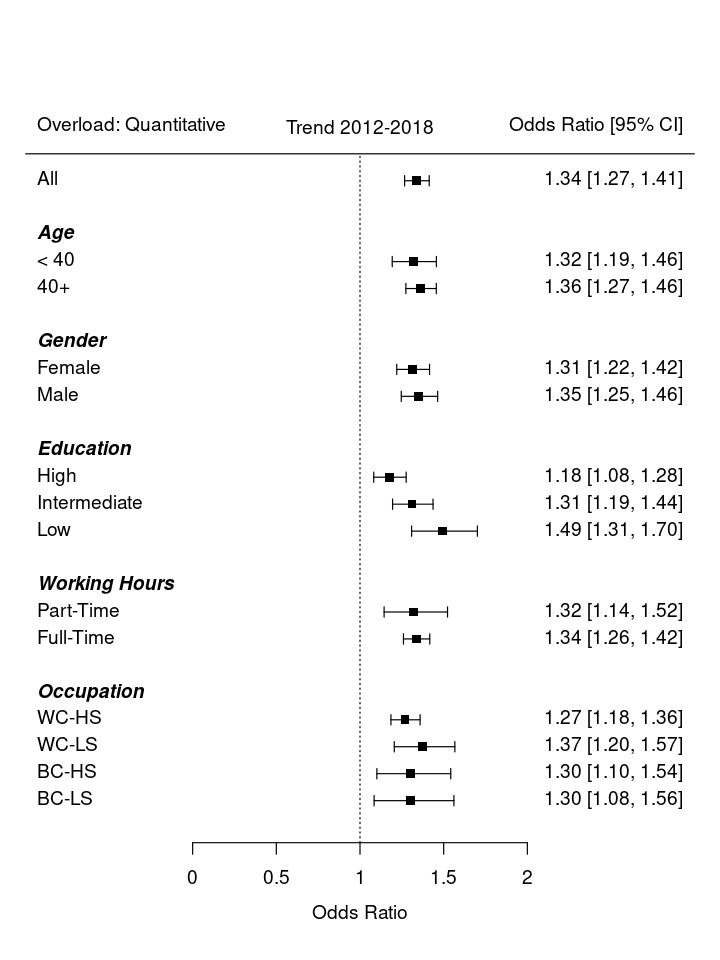


*Notes.* Depicted are the odds ratios for time period predicting work overload controlling for age and gender via logistic regression using weights comparing 2006 vs 2012 and 2012 vs 2018. For each subgroup a separate logistic regression analysis was conducted. WC-HS = White-Collar High Skilled; WC-LS = White-Collar Low Skilled; BC-HS = Blue-Collar High Skilled; BC-LS = Blue-Collar Low Skilled

Table A1. *Effect Size of Sociodemographic Differences in Work Overload for Each Year*

| **Variable** | **2006** | | **2012** | | **2018** | |
| --- | --- | --- | --- | --- | --- | --- |
|  | Qualitative Overload | Quantitative  Overload | Qualitative  Overload | Quantitative  Overload | Qualitative  Overload | Quantitative  Overload |
| Age | 0.001 | 0.006 | 0.003 | 0.010 | 0.008 | 0.001 |
| Gender | 0.003 | 0.030 | 0.005 | 0.055 | 0.001 | 0.055 |
| Education | 0.022 | 0.039 | 0.037 | 0.041 | 0.039 | 0.025 |
| Working Hours | 0.014 | 0.075 | 0.016 | 0.076 | 0.023 | 0.078 |
| Occupation | 0.010 | 0.046 | 0.013 | 0.047 | 0.010 | 0.042 |

*Notes.* Depicted are the Cramer’s *V* effect sizes for the sociodemographic comparisons regarding work overload across time periods.
